# Let it grow—the open market solution to marijuana control

**DOI:** 10.1186/1477-7517-11-32

**Published:** 2014-11-18

**Authors:** Jon Gettman, Michael Kennedy

**Affiliations:** Shenandoah University, 1460 University Drive, Winchester, VA 22601 USA; 250 West 57th Street, Suite 920, New York, NY 10107 USA

**Keywords:** Cannabis, Decriminalization, Drug traffic, Government policy, Drug legalization (United States), Drug control (economic aspects), Drug policy, Legalization markets, Marijuana, Regulation

## Abstract

This commentary evaluates regulatory frameworks for the legalized production, sale, and use of marijuana. Specifically, we argue that the primary goal of legalization should be the elimination of the illicit trade in marijuana and that maximizing market participation through open markets and personal cultivation is the best approach to achieving this goal. This argument is based on the assertion that regulatory models based on a tightly controlled government market will fail because they replicate the fatal flaws of the prohibition model. This commentary argues that an examination of the reasons for prohibition’s failure—to wit, the inability of government to control the production of marijuana—completely undercuts the basic premise of a tightly controlled market, which depends on the ability of the government to control production. The public interest would be better served by an effective regulatory framework which recognizes and takes advantage of competitive market forces. This analysis argues that reducing teenage access to marijuana requires the elimination of an overcapitalized illicit market. Further, it asserts that this goal and maximization of tax revenue from a legal marijuana market are mutually exclusive objectives.

## Commentary

### Background

This article presents the case for legalizing marijuana by way of a wide-open commercial, competitive market including the allowance of small-scale cultivation for personal use.

Big changes are occurring in the marijuana laws of the United States. These changes are driven primarily through voter initiative campaigns designed to bypass state legislatures, garner majority public support, and accelerate a decades-old trend of state departure from the prohibition regimen of federal law. Outright legalization of the use and commercial trade in marijuana has joined decriminalization, prosecutorial discretion, conditional discharge, and medical marijuana exemptions in the catalogue of state tactics to opt out of the federal criminalization of marijuana sales and possession and the classification of marijuana as a drug similar to heroin in terms of individual and social harm.

So, now that legalization is on the table, so to speak, what sort of legalization is best for the public interest? The answer is simple: the sort that works where the abandoned policy of prohibition has failed. Many academics do not seem to understand that simple point. The problem is the issue of control, as in drug control, and the reality of current policy is that there is no control. That is why states have been and will continue to opt out of the rigid federal prohibition. Some academics and policy officials are now advocating new approaches based on a desire to institute tight controls, conveniently overlooking that this is the exact approach that created the current mess. This article will review that perspective, expand on what the lesson from prohibition should be, and apply this lesson to defend open market solutions to the problems and challenges of creating effective regulations for a legal marijuana market.

The debate over marijuana policy is changing from whether to legalize marijuana to how to regulate a legal market. Criticism of marijuana prohibition is widespread, and there is broad consensus among critics that it has failed and why it has failed. Critiques are often based on the persistence of wide and unchanged access to marijuana (especially to teenagers), prohibition’s failure to provide medical access, racial disparities in marijuana possession arrests, and the costs of arrests to both individuals and society [1–3]. A considerable amount of discussion has addressed the clash between state-level reforms, such as medical marijuana laws, and the ongoing federal prohibition in the United States [4–13]. This discussion often focuses on a) ways to reconcile state reforms with federal prohibition and b) the benefits of policy innovation at the state level.

Legalization of marijuana at the state level in the United States in Colorado and Washington, at the national level in Uruguay, and the likelihood of additional state action in the United States has generated a new round of discussion. The focus has shifted to the objectives, dynamics, potential features, and other critical issues concerning regulatory frameworks for a legal marijuana market. Examples of this discussion are found in articles by Caulkins et al. [14] and Room [15], along with additional commentary by other authors, in the journal *Addiction* and a panel discussion between Mark Klieman, Alison Holcomb, Sue Rusche, and Jonathan Rauch sponsored by the New American Foundation [16].

An initial approach rests on the premise that strict controls on marijuana are justified by public health concerns. Cohen and McGowan provide a straightforward synopsis of popular thinking on this subject. They assert that the goals of marijuana legalization should be controlling consumption, eliminating the black market, and generating state revenues [17]. The best way to achieve these objectives, they and others theorize, is through government monopoly [16, 18]. The rationale is that "keeping marijuana out of the private marketplace allows states more control in their vital role of limiting use by minors [17]." Cohen and McGowan support their theory by evoking the spectre of "Big Cannabis" which, like "Big Tobacco," will advertise and market marijuana to increase consumption and stimulate teen use. State-run stores, according to this proposition, have no incentive to promote sales [18].

Support for government monopoly is bolstered by the fear that a dramatic drop in marijuana prices will lead to increased consumption [14]. Taxes, then, should be used to inflate marijuana prices, keeping them near or just below current levels, to discourage consumption and maximize tax revenues [16, 18–20].

What is missing from these discussions, first of all, is a realistic consideration of marijuana cultivation, particularly personal or home cultivation. There are two notable exceptions to this omission. Reuter observes that this may be the only way to curtail commercialization but observes that this would deny states tax revenue [21]. Caulkins et al. concede there are many arguments for allowing home cultivation, including diverting market share from commercialized interests, sharing and gift giving, and fostering nonprofit cooperative efforts [14]. If market forces can avert a price collapse, an important share of the market could be seized by personal cultivation. On the other hand, if prices do collapse, personal cultivation would be limited to hobbyists. Caulkins et al. also express concern, however, that allowing home cultivation would make it harder to regulate commercial production and distribution [14].

What is also missing from these discussions is a general awareness or recognition of how detached scholarly analysis of marijuana control efforts has been over the last several decades. This can be evidenced by a general evaluation of the accuracy of the data that informs such analysis and a specific review of data relevant to a minimal assessment of the impact of control efforts on participation in the production and supply side of the market.

Regarding the accuracy of data, three revelations tell the story, and the story is that whatever the government thinks it knows about marijuana use and cultivation is usually discovered to be only the tip of the iceberg. First, in 1981, the DEA estimated that 1,200 metric tons of marijuana was produced in the United States. In 1982, they seized 1,653 metric tons. "Therefore, the program shows that in 1982, 38% more domestic marihuana was eradicated than was previously believed to exist [22]." Second, in 2002, the National Survey on Drug Use and Health (NSDUH) revised its data collection procedures and increased their estimate of annual marijuana users from 21.1 million (as reported in the 2001 survey results) to 25.7 million [23, 24]. Third, after reporting from 1998 to 2000 that domestic marijuana production was 3,500 metric tons [25], the Office of National Drug Control Strategy reported in February 2003 that US production was actually more than 10,000 metric tons [26]. This is a recurring issue. A 2013 RAND study estimated that the amount of marijuana consumed in the State of Washington (120 to 175 metric tons) was considerably greater than the earlier estimate of the Washington Office of Financial Management (85 metric tons) due to underreporting in prior survey data [27].

Realistic evaluations of drug control efforts should look at market participation, and this means producers and sellers more so than users. Here, the available data, tip of the iceberg or not, reinforces the conclusion that existing efforts are unsatisfactory. Consider the following. According to Uniform Crime Reporting Program data, there were 67,485 arrests for marijuana sales in 1990, 74,208 in 2000, 87,759 in 2010, 76,404 in 2012, and an average of 76,266 from 1990 to 2012 [28]. This is significant not so much with respect to program output or deterrence issues as it is an indication of persistent market participation. Indeed, the NSDUH provides estimates of the number of people who sell illegal drugs in the United States; from 2003 to 2012, there were an average of 4,623,352 people selling illegal drugs annually [29]. This includes all drugs, but given that marijuana is the most popular illegal drug, it is another good indicator of the extent of market participation. Also, the Drug Enforcement Administration seized 3,347 indoor marijuana grow operations in 1993 (with 290,452 plants), 2,678 in 2003 (with 223,183 plants), 3,713 in 2007 (with 434,728 plants), and 2,596 grow rooms in 2012 (with 302,377 plants) [30]. These data indicate strong, persistent, and consistent levels of market participation in terms of production and sales. The NSDUH also provides data on the number of personal-use marijuana cultivators, which has increased dramatically from 206,335 in 2003 to 477,028 in 2012, an increase of 131% [29].

The evidence above indicates that a) the market in marijuana is consistently found to be larger than previously believed to exist, b) there is widespread and consistent participation in production and sales, and c) involvement with personal cultivation of marijuana is skyrocketing. Marijuana prohibition of production and distribution is unenforceable. Any assertion that tight control of a legal market through limiting participation and artificial price inflation by way of taxation can be successful where prohibition failed is dubious at best and flies in the face of the historical experience and empirical evidence of the last generation.

### An alternative perspective

Marijuana’s prospective legalization should be viewed simultaneously as a remedy to the failures of prohibition and as a means to achieve important public policy objectives. Ethan Nadelmann, Executive Director of the Drug Policy Alliance, instructively notes that

"Any model for legally regulating cannabis production and distribution must be compared not just with an ideal scenario but with the realities of contemporary cannabis prohibition" [31].

Public policy has much in common with scientific theory, especially in terms of evaluation. Kuhn argues that theories are best subject to the standards that existed when they were first proposed [32]. In this manner, Kaplan provides useful guidance for assessing why marijuana prohibition has failed in the form of two crucial observations:

"[A]n important factor in the success or failure of any method of drug control is the degree to which the users want the drug… [and] the technology of drug production and consumption is an important factor in the success or failure of a drug-control measure. Where the technology of drug production and distribution is not difficult to overcome, drug control will be very difficult" [33].

Marijuana, as a commodity for production, has unique attributes that distinguish it from alcohol and tobacco. It is relatively easy to grow and does not require industrial processing. Marijuana can be produced anywhere by just about anyone. It is grown throughout the country, in backyards, closets, attics, basements, and warehouses. While little technology is needed to grow marijuana, ample technology to maximize production and yield are widely, legally, available. This is a considerable factor in why prohibition has failed to control the production of marijuana. This will also be a considerable factor in the success or failure of any alternative regulatory regime.

With respect to public policy, the purpose of regulation should be to enhance protective factors and mitigate risk factors. These objectives should take precedence over other potential objectives, specifically maximizing tax revenue. The necessity of many regulatory measures is widely recognized. These include age and identification requirements for purchase, record-keeping, potential purchase limitations, advertising and marketing restrictions, health warnings and packaging requirements, and labelling standards [1, 14, 16].

While it may seem counterintuitive, the ubiquitous nature of marijuana production can be a benefit rather than a threat to achieving public policy objectives. The concern with commercialization would be better expressed as concern with the activity of an oligopolistic market rather than a competitive one. Indeed, the current market in tobacco is an oligopoly [34], and generic products are viewed as an industry killer [35]. Home cultivation of marijuana should likewise be viewed as an oligopoly killer, consistent with the observations of Caulkins et al. [14] and Reuter [27].

In addition to production, the other key element in evaluating regulatory frameworks concerns Kaplan’s first point, the degree to which users wish to use the drug. Consideration of consumer interests should be a key component of any discussion about regulatory objectives. There are three important considerations. First, given the failure of the compulsory prohibition, voluntary compliance will be required for regulations to be successful. Second, consumers have two major and self-evident complaints about prohibition. They resent being subject to arrest and other sanctions. And, like any consumers, they do not like high prices. The third important consideration, discussed below, regards the impact of regulations in terms of market forces and how they influence competitive behavior and the pursuit of profits.

Porter had detailed how five forces affect market competition and competition over profits and thus determine operational strategies [36]. Public policy can influence these strategies by influencing the nature of these competitive forces. The five competitive forces that determine the structure of market are 1) availability of substitute products, 2) ease of market entry, 3) leverage of suppliers, 4) leverage of consumers, and 5) rivalry among competitors. The influence of each force varies from market to market, and strategy is usually a response to the most influential forces in a particular market. Government regulation typically has its greatest impact on ease of entry into a market and by its impact on supply, since regulation impacts price. In a regulated marijuana market, given the widespread availability of production technology, consumers are also potential suppliers. This dual role gives them considerable influence over market activity, influence comparable in importance to that of regulatory provisions and the rivalry of competitors.

The influence of marijuana consumers will be an important determinant in the success of failure of any regulatory framework for marijuana. Porter explains that consumers, or buyers of products, act naturally in their own interests to force down prices and bargain for quality or services. The power of buyers depends on various circumstances, which include high prices relative to buyer income, a market of standard or undifferentiated products, low switching costs for changing suppliers, credible capabilities for backward integration (meaning, self-supply), and access to market data [36]. Limiting market access and maintaining artificially high prices will enhance the power of buyers; they will seek other sources and/or grow marijuana for themselves and others.

This has not been adequately recognized in prior discussions of this issue. There is, consequently, a trade-off between maximizing government revenue and reducing black market production and sales. The trade-off has been recognized, but the likely persistence and magnitude of unlicensed cultivation in a strictly controlled market have been overlooked.

### The value of frameworks

Marijuana’s legalization raises numerous critical issues for investigation and discussion, and articles such as this raise far more questions than they can put to rest. This highlights, though, the need to examine this topic in terms of regulatory frameworks and the general principles they incorporate. The discussion above is meant to introduce new elements to the consideration of prospective regulatory policy for marijuana such as a) the reason prohibition has failed, b) the empirical limits on government’s capacity to impose controls, and c) a more useful perspective or model for understanding the forces affecting the market. There is a simplifying assumption apparent here and one that often gets lost in academic review and/or policy analysis. This is the Jeffersonian proposition that people affected by government action should have a voice and a role in its formulation. In other words, the creation of regulations for the legalization of marijuana require input from and support of the producers and consumers it will regulate to ensure the voluntary compliance required to make new policies successful. The value of frameworks, then, is that they organize critical issues and provide interested parties with clear choices.

### The three models and their impact on competition and price

The current regulatory model for marijuana is prohibition, in which criminal law prohibits manufacture, distribution, and possession of marijuana, and the resulting illegal market is regulated solely through the tool of risk assessment. Anyone willing to bear the risk of criminal prosecution may enter and participate in the market. The illegality of the market acts as price support. This is often explained in terms of a risk premium. However, it can also be understood as the result of an absence of consumer protections; sellers are free to overcharge consumers, who have no recourse. In other words, price fixing is also a characteristic of the black market. The result is that competition is great and prices are high.

The second type of model under consideration provides for legalization of marijuana and will be referred to here as the interventionist model. This model has two forms: A government monopoly (such as with alcohol sales in 18 states) and a market with access determined by limited government licenses (such as the current legal market for marijuana in Washington state). It can be characterized in terms of limited market access, high prices, low levels of competition among merchants, and high levels of tax revenue. This approach is advanced in one form or another by Brannon [20], Cohen and McGowan [17], Klieman, Rauch, and Rusche in Glastris [16] Klieman [18], and Pederson [37]. In either form, the result is that competition will be low and prices will remain high.

The third model, as proposed here, also provides for legalization but instead is based on an open, competitive market solution. In this model, aside from some perfunctory regulatory requirements, market entry is unrestricted and there will be a large number of producers; essentially, anyone or any firm that is able to enter the market and willing to bear the risks may participate. This includes, most importantly, individuals who wish to grow marijuana for their personal use and/or small-scale transfers to their friends and associates. This level of competition will result in substantially lower prices than the prices that exist in the current market or would exist under the interventionist model. The result is that competition will be high and prices will be low.

### The three models and their primary objectives

There are three popular theoretical justifications offered for prohibition. The first is classic deterrence theory tied to criminal and other sanctions. Punishment is meant to provide specific deterrence to those prosecuted and general deterrence to the public. There are several flaws with this rationale. Research on deterrence suggests that it has little impact on expressive (rather than instrumental) crimes, on high-committed offenders, and on private rather than public crimes [38]. Another issue is certainty of arrest and severity of punishment. Neither concern has much relevance to the recent history of marijuana law enforcement in the United States. The second rationale for prohibition, in terms of criminological theory, is social learning theory, in which school authorities, police, mass media, and other important influences affect learned behavior through promotion of rewards or punishments [38]. The third justification for prohibition is that high prices discourage use. It is important to note that the government and illicit merchants act in collusion under this model, as high prices are widely seen as a deterrent to drug us. Illicit merchants overcharge for their products, fulfilling government policy of discouraging wider use through artificially inflated prices.

In the interventionist model, the government, in effect, nationalizes the illegal market. The objective is to keep prices high but to lower the number of vendors and reallocate the transfer of wealth from criminal actors to the government and its licensees. The rationale for this model is threefold. First, the price of marijuana must be kept high in order to discourage consumption. Second, commercialization of marijuana must be prohibited in order to prevent commercial inducements to the number of consumers or the amount of consumption. Third, this market structure will maximize government tax revenue.

The primary objective of the competitive model is more modest. The open market model seeks to destroy the illegal market through the process of creative destruction. This is a widely recognized economic doctrine introduced by Schumpeter in which new combinations of goods and services divert capital from existing markets to new market, and thus, the creation of new markets destroys the old ones [39]. In this context, an open competitive market for marijuana’s production and distribution will a) reduce and eliminate participation in the illicit market and b) provide a counterweight to monopolistic or oligopolistic commercial excess.

### Role of government

In the prohibition model, the government seeks to exert control through the use of a single tool. This is often conceived in terms of criminal sanctions, but in practice and in terms of market forces this is really an attempt to control the market by determining entry costs. The notion that criminal penalties and law enforcement can curtail this activity has already been disproven through historical experience.

The interventionist model seeks to influence the market through the use of three tools: central planning, tax policy, and consumer protection regulations. Central planning, it is argued here, is problematic when it comes to a commodity so easily and commonly produced without regard for government policy. Tax policy will be addressed below. Consumer protection regulations should be a component of any regulatory policy for marijuana and are not at issue here.

The competitive open market model seeks to influence the market through the use of competitive forces and, like the interventionist model, consumer protection regulations. Incorporating existing producers into the market through open access and personal cultivation not only co-opts participation in the illegal market but also enhances competition. A competitive market has many of the same virtues of a large republic, calling to mind James Madison’s admonition in Federalist #10 that multiple factions preserve liberty through what in modern times has been referred to as establishment of a balance of power [40]. This principle also applies to competitors in economic markets. In political markets, pluralism protects freedom. In economic markets, pluralism protects consumers. In both markets, pluralism protects the public interest.

The new regulated market must incorporate rather than replace production from the current market. Many current producers fear a corporate takeover of marijuana production that would force them out of the business [41–43]. But if the objective of a regulated market is to eliminate or reduce the scope of the illegal market, there needs to be a place in the new market for old producers; otherwise they may continue production and undermine the regulated market in much the same way as they undermine prohibition. This argues against prohibiting individuals with convictions for marijuana production or distribution crimes from participating in the new, legalized market.

### Tax revenues

The prohibition model does not provide tax revenue. Instead of benefits, it creates costs. On the other hand, the interventionist model seeks to maximize tax revenue, justifying this on the premise of reducing consumption, but carries the risk of encouraging out-of-market behavior. The idea of using tax policy to maintain prices for marijuana at or near current levels is not unprecedented, as attempts were made to apply this policy to alcohol after the end of prohibition. The result is counterproductive. For example, an analysis of the top activities of 162 soldiers of New York mafia families from 1950 to 1963 indicates that 11% were involved in evasion of alcohol taxes through bootlegging or moonshining activities [44]. According to Hortis,

"Perhaps most surprising was the wise guys’ continued role in illegal alcohol sales after the repeal of prohibition in 1931. This was another example of how over-regulation fostered organized crime. Through the 1950s, the federal excise tax on whiskey was extremely high at $10.50 a gallon. (If the excise tax had kept up with inflation, it would be $90 a gallon in 2013 dollars instead of its current rate of $13.50) State and federal regulations further drove up the price of booze" [45].

To maintain current prices through taxation requires tax rates significantly higher, with respect to the costs of production, than those applied now to alcohol. Given the widespread recognition that marijuana is much less dangerous to use than alcohol, this begs the question as to how taxing it at a much higher rate can be justified. Furthermore, as in the example above, such overregulation creates classic opportunities for criminal profiteering.

Small-scale production and trade in marijuana are not significant threats to tax revenue for two reasons. First, there will not be substantial profits to be realized from such activity because of relatively low prices. Second, most consumers will be attracted to the commercial market anyway. There will not be a high volume of untaxed commerce. Furthermore, the lack of a significant profit potential will mitigate against sales to minors and against sales by minors to their peers (see below). A large number of competitors will marginalize any benefits from marketing to minors, since there is no guarantee or certainty that such efforts will have significant impact on the marketer’s own profits. Sales in the legal market will be diffused over a large number of producers. Finally, consumers will benefit from significant consumer savings compared to the prior prohibition framework, enhancing their voluntary participation and political support for this approach.

### Impacts, illegal profits, and teenage marijuana use

Prohibition produces a highly capitalized black market with moderate levels of competition in terms of price, quality, and service. While public health, theoretically, benefits from discouragement of use, this benefit is offset by widespread availability and unsustainable costs. Two primary costs are the lack of tax revenue and the overall costs of law enforcement. Additional costs include widespread teenage access to a market without age restrictions on purchases and the availability of other illicit drugs to customers of all ages. Social costs include various inequities such as racial and other disparities in arrest rates. Costs to the consumer include the potential costs of arrest and imprisonment, other social sanctions such as loss of employment due to drug testing or arrest, and the government-sanctioned transfer of wealth from consumers to illicit market participants.

Prohibition encourages participation in the illegal market through artificially created profit potential. High prices attract entrepreneurs; potential profit stimulates production and distribution. In 1992, according to the NSDUH, at least 1.1 million individuals sold illegal drugs. In 2002, this number increased to 3.5 million, and in 2012, it increased again to 4.7 million [46]. Often overlooked in discussion of teenage marijuana access is how many teens sell illegal drugs. In 1992, there were 313,000 teens selling drugs, increasing to 1 million in 2002, and falling to 680,000 in 2012. These statistics suggest that our current policy fails in part because prohibition makes it profitable for teenagers to sell marijuana [29].

The interventionist model suffers from the same constraints as prohibition. The inability to enforce production controls is why prohibition has failed and legalization is being considered. Legal market success will rely on voluntary compliance by current consumers and producers; this will not result by imposing a framework on the public. The government’s ability to design, operate, and supervise a multi-billion dollar market is questionable on practical and philosophical grounds. On a practical basis, government regulation routinely faces the risks of regulatory capture [14, 47] and revenue addiction, making regulators and politicians not only promoters but targets for corruption as well [47]. This will be a problem for any regulatory scheme. The stricter the controls, the more likely corruption, incompetency, or both will result. Woodruff, from the *National Review*, observes that

"People who sell marijuana legally have to deal with a lot of the same annoying, unsexy problems that other businesses face, including cronyism and incompetent bureaucratic oversight" [46].

On a philosophical level, there is considerable opposition in some circles to regulatory authority that allows government to pick winners and losers, and to the concept of central planning in general. The interventionist model is, in effect, a proposal that bureaucratic nonspecialists service a market of resentful consumers and successfully compete with an up and running, unregulated, and profitable illicit market. The idea that the solution to the ills of marijuana prohibition is to nationalize the market through a government takeover fails to take into account the very reasons for the existence of the problem it seeks to resolve.

Competition will be limited in this model because the rationing of licenses guarantees strong market shares for licensees. There will not be as great of an incentive to compete in terms of price, quality, and service in order to make a profit. This lack of responsiveness to consumers, along with high prices, will result in continued (and presumably) illicit home and small-scale production. Because the cost of producing marijuana is relatively low, whatever the fixed price of marijuana is, it will be undercut by illicit producers seeking profits. Consequently, black market opportunities will persist. Teenagers will continue to have access to marijuana through teen-to-teen sales and overall black market availability. Finally, consumers will not benefit from consumer savings produced by a drop in the price of marijuana.

The competitive open market model has advantages not enjoyed by the other alternatives. Several observers have already noted increasing differentiation in the various interests in favor of marijuana’s legalization. This means, using Madison’s terms, the emergence of competing factions with overlapping and at times conflicting interests. Ethan Nadelmann, executive director of the Drug Policy Alliance, told *Rolling Stone* that "the people who may come to dominate this [new] industry are not necessarily the people who are a part of the movement" [19], p. 35. Mark Klieman told the *Washington Post* that the public interest and the goals of the legalization movement are similar; it is the goals of the commercialization model that clash with the public interest [48]. Caulkins cautions that in any legal regime, there will be stakeholders with a vested interest in preserving their livelihood [49]. The way to control these competing interests is to enhance, rather than restrict, competition.

### Conclusions

What is missing from most analyses is recognition that prohibition failed through an inability to control production. Betsy Woodruff from the *National Review* put it succinctly: "A big part of the problem is that the federal government has a law that it can’t enforce" [46]. Any new regulatory regime for marijuana must pass the enforcement test. Continued prohibition of personal cultivation is unenforceable. This is not the only problem with using public policy to prop up the price of marijuana, regardless of rationale or objective. High prices for marijuana provide production incentives in a market for which there is not a viable way to restrict production by regulation or criminal sanction.

Under any of these frameworks, the key question with respect to public health is how to protect vulnerable populations. Danovitch provides a good description of the problem:

"Like most drugs, marijuana has some potential benefits and some potential risks… most of the risks associated with marijuana are moderate in severity, the prevalence of marijuana use means that a sizeable minority of the population is likely to experience some adverse effects. Furthermore, three populations are particularly at risk for the adverse effects of marijuana: youth, individuals with mental illness, and pregnant or breastfeeding women" [6], p. 107.

Each of these populations has unique features that present public health challenges. Prohibition has not been able to provide protection for any of them. One of the most compelling rationales for legalization is the need to protect these at-risk populations more effectively. Commercialization may complicate efforts to discourage teenagers from using marijuana, but the real problem there is what Caulkins has referred to as "the intrinsic difficulty of changing teens’ behaviour" [49]. Klieman is reconciled to the persistence of teenage marijuana use even with a government monopoly, noting that it would be better for teens to get marijuana through quasi-legal sources than the black market [16], where they would presumably have access to dangerous illegal drugs such as opiates, cocaine, and methamphetamine. Individuals with mental illness and pregnant or breastfeeding women are problematic, just as with teenagers’ education, and prevention and sometime interventionist approaches to counter potentially risky behavior are required. In a competitive market, there are more stakeholders and thus more incentives to cooperate, fund, and otherwise support such measures.

The justification for the interventionist model (as for prohibition) is that marijuana must be costly in order to discourage use. This assertion must be subjected to critical examination. Certainly, it is consistent with basic economic logic. However, it should be assessed in light of additional issues such as past performance and experience, the impact of price on current usage patterns, the influence of potency and tolerance on current use, and other relevant factors. Marijuana is widely available under current conditions. One could argue that high prices for marijuana have not provided a significant constraint on its popularity.

Also, high prices may even, in some cases, facilitate heavy use of marijuana. For example, under current market conditions, heavy users have an incentive to grow and sell marijuana in order to subsidize or cover the cost of their own use. The relationship between substance use and drug dealing among juveniles is well documented [50]. Selling drugs is a sensible and logical way to both gain access and reduce cost.

For the purposes of this critique of a tightly controlled market, it will suffice to acknowledge that under legalization, any form of legalization, the public will have greater access to marijuana and that overall usage will increase. Nonetheless, the benefits of legalization are likely to offset or exceed such an outcome. The benefits of legalization with respect for public safety are summarized well by Roffman:

"I believe that prohibition’s track record in protecting public health and public safety has been seriously deficient. Moreover, inequities in prohibition’s implementation make evident it has been fundamentally flawed in terms of social justice. When the evaluation data begin to become available over the coming years, among the outcomes I hope to see, in contrast with what we have witnessed prior to legalization, are: fewer young people initiating marijuana use prior to age 21, fewer students struggling with school performance as a consequence of marijuana use, a smaller percentage of users becoming marijuana dependent, more of those who become dependent receiving effective treatment, fewer traffic accidents in which marijuana smoking is a contributing factor, and more accurate knowledge held by the public concerning marijuana’s effects on health and behaviour" [51].

The production issue makes it difficult if not impossible to establish a closed market. This is one of the specific goals of the Controlled Substances Act, and the failure to achieve this is one of the most significant failures of the prohibition model. It would, in due course, contribute to the failure of the interventionist model as well. This leaves policy makers with a dilemma. They must choose between an effective regulatory framework that eliminates the black market and a government takeover that produces significant tax revenues but with many of the same costs and externalities as prohibition.

Tax revenue should not be a primary objective of marijuana’s legalization. It should, along with economic development, be viewed as a subsidiary benefit. The legalization of cannabis will produce considerable economic benefits in the form of new industries and new commodities. Legalization will produce jobs, incomes, and tax revenue. Lower prices will also reallocate consumer savings, diverting money now spent on marijuana to other forms of economic activity.

More importantly, a competitive market offers greater benefits with respect to public policy, especially the reduction of teenage use through restricted access and the elimination of profit incentives for teen-to-teen sales. Teenagers will continue to use marijuana, but as Roffman points out, the overall social environment under legalization, with respect to public health, will be far better than it is under prohibition. A competitive market provides the opportunity to maximize those benefits. Most marijuana users, the majority of the subculture associated with marijuana use, are resistant to a corporate oligopoly taking over control of marijuana production and distribution in the United States. It is time to enlist this community in the pursuit of the public interest. To this end, it is recommended that the ongoing discussion over an appropriate regulatory framework be expanded to include the issue of corporate social responsibility and the extent to which this can be augmented by many of the shared values of the existing subculture of marijuana users.

The reason marijuana legalization is gaining in popularity is recognition that prohibition has failed to control the market. The solution to the challenge of creating a legal, regulated market in marijuana is to let it grow. Let the market grow, an open, competitive market with high levels of participation and lower prices.

## Authors’ information

JG is an assistant professor in Criminal Justice at Shenandoah University. MK is a practicing criminal defense attorney and a Director of the Trans-High Corporation, publisher of *High Times*, and the High Times Growth Fund.

## References

[1] Blumenson E, Nilsen E (2009). No rational basis: the pragmatic case for marijuana law reform. Va J Soc Pol’y & L.

[2] King R, Mauer M (2006). The war on marijuana: the transformation of the war on drugs in the 1990s. Harm Reduct J.

[3] Pudney S (2010). Drugs policy: what should we do about cannabis?. Econ Policy.

[4] Carcieri M (2012). On the medicinal-recreational distinction in cannabis law. Denver U Law Rev.

[5] Cohen P (2010). Medical marijuana 2010: it’s time to fix the regulatory vacuum. J Law Med Ethics.

[6] Danovitch I (2012). Sorting through the science on marijuana: facts, fallacies, and implications for legalization. McGeorge Law Rev.

[7] Kamin S (2012). Medical marijuana in Colorado and the future of marijuana regulation in the United States. McGeorge Law Rev.

[8] Mikos A (2011). A critical appraisal of the department of justice’s new approach to medical marijuana. Stanford Law Policy Rev.

[9] Mikos R (2013). Preemption under the controlled substances act. J Health Care Law Policy.

[10] O’Keefe L (2013). State medical marijuana implementation and federal policy. J Health Care Law Policy.

[11] Shechtman M (2012). Joint authority? The case for state-based marijuana regulation. Tennessee J Law Policy.

[12] Simoni-Wastila L, Palumbo F (2013). Medical marijuana legislation: what we know - and don’t. J Health Care Law Policy.

[13] Tutro J (2013). States are making their own decisions regarding whether marijuana should be illegal: how should the federal government react?. Tennessee J Law Policy.

[14] Caulkins J, Kilmer B, Maccoun R, Pacula R, Reuter P (2012). Design considerations for legalizing cannabis: lessons inspired by analysis of California’s Proposition 19. Addiction.

[15] Room R (2014). Legalizing a market for cannabis for pleasure: Colorado, Washington, Uruguay and beyond. Addiction.

[16] Glastris P, (moderator) (2014). The Corporate Takeover of Marijuana: How Not to Make a Hash Out of Marijuana Legalization.

[17] Cohen E, McGowan R (2012). Grass is always greener when it’s legal: policies for state regulated marijuana. The Economists Voice.

[18] Klieman M (2014). How Not To Make a Hash Out of Cannabis Legalization.

[19] Barcott B (2014). A tale of two drug wars. Rolling Stone.

[20] Brannon I (2013). Legalizing marijuana: money over minds. Regulation.

[21] Reuter P (2014). The difficulty of restricting promotion of legalized marijuana in the United States. Addiction.

[22] Cannabis Investigations Section (1982). Domestic Marihuana Eradication/Suppression Program, 1982.

[23] Substance Abuse and Mental Health Services Administration (2014). 2001 National Household Survey on Drug Abuse.

[24] Substance Abuse and Mental Health Services Administration (2014). 2002 National Survey on Drug Use and Health.

[25] Office of National Drug Control Programs (2002). National Drug Control Strategy – 2002.

[26] Office of National Drug Control Programs (2003). National Drug Control Strategy – 2003.

[27] Kilmer B, Caulkins JP, Gregory M, Linden D, MacCoun RJ, Pacula RL (2013). Before the Grand Opening, Measuring Washington State’s Marijuana Market in the Last Year Before Legalized Commercial Sales.

[28] Federal Bureau of Investigation: *Uniform Crime Reporting Program Data: County-Level Detailed Arrest and Offense Data, [1990–2012]*. Ann Arbor: Inter-university Consortium for Political and Social Research [distributor];

[29] Center for Behavioral Health Statistics and Quality: *National Survey on Drug Use and Health, [1991–2012]*. Ann Arbor: Inter-university Consortium for Political and Social Research [distributor];

[30] Domestic Cannabis Eradication Suppression Program: *Number of Marijuana Plants Eradicated and Seized*. Albany: Sourcebook of Criminal Justice Statistics; Table 4.38, various years. [ http://www.albany.edu/sourcebook/]

[31] Nadelmann E, Gutwillig S, Davies J (2012). Additional considerations. Addiction.

[32] Kuhn T (1977). The Essential Tension: Selected Studies in Scientific Tradition and Change.

[33] Kaplan J, Blum R, Bovet D, Moore J (1974). Classification for legal control. Controlling Drugs, International Handbook for Psychoactive Drug Classification.

[34] Debertin D (2001). Corporate strategy in the tobacco manufacturing industry: the case of Philip Morris. Rev Agric Econ.

[35] Porter M (1985). Competitive Advantage: Creating and Sustaining Superior Performance.

[36] Porter M (1980). Competitive Strategy: Techniques for Analyzing Industries and Competitors.

[37] Pedersen W (2014). The powerful mix of capital and cannabis culture. Addiction.

[38] Barkin S (2012). Criminology: A Sociological Understanding.

[39] Schumpeter J (1950). Capitalism, Socialism, and Democracy.

[40] Hamilton A, Madison J, Jay J (1961). The Federalist Papers.

[41] Abramsky S (2010). Altered state. Nation.

[42] Gwynne K (2013). Will medical pot survive?. Nation.

[43] Lawson R, Nesbit T (2013). Alchian and Allen revisited: law enforcement and the price of weed. Atlantic Econ J.

[44] Hortis CA (2014). The Mob and the City, The Hidden History of How the Mafia Captured New York.

[45] Hortis CA (2014). The Mob and the City, The Hidden History of How the Mafia Captured New York.

[46] Woodruff B (2013). Rocky Mountain High: Colorado experiments with marijuana. National Review.

[47] Kinsley M (2013). Joint committee. New Republic.

[48] Terris B (2014). Big Pot rising: the marijuana industry’s first full-time lobbyist makes rounds on Capitol Hill. Washington Post.

[49] Caulkins J (2012). A future of legalized marijuana?. World Future Rev.

[50] Floyd LJ, Alexandre PK, Hedden SL, Lawson AL, Latimer WW, Giles N (2010). Adolescent drug dealing and race/ethnicity: a population-based study of the differential impact of substance use on involvement in drug trade. American J Drug Alcohol Abuse.

[51] Roffman R (2013). Legalization of marijuana: unraveling quandaries for the addiction professional. Frontiers in Psychiatry.

